# Parent-Child Interaction Using a Mobile and Wireless System for Blood Glucose Monitoring

**DOI:** 10.2196/jmir.7.5.e57

**Published:** 2005-11-21

**Authors:** Deede Gammon, Eirik Årsand, Ole Anders Walseth, Niklas Andersson, Martin Jenssen, Ted Taylor

**Affiliations:** ^2^Oregon Research InstituteOregonUSA; ^1^Norwegian Centre for TelemedicineTromsøNorway

**Keywords:** home blood glucose monitoring, diabetes, self-management, parent-child relations, wireless communication, mobile phones, pediatric psychology

## Abstract

**Background:**

Children with type 1 diabetes and their parents face rigorous procedures for blood glucose monitoring and regulation. Mobile telecommunication systems show potential as an aid for families’ self-management of diabetes.

**Objective:**

A prototype designed to automatically transfer readings from a child’s blood glucose monitor to their parent’s mobile phone was tested. In this formative stage of development, we sought insights into the appropriateness of the concept, feasibility of use, and ideas for further development and research.

**Methods:**

During four months, a self-selected sample of 15 children (aged 9 to 15 years) with type 1 diabetes and their parents (n = 30) used the prototype approximately three times daily. Parent and child experiences were collected through questionnaires and through interviews with 9 of the parents.

**Results:**

System use was easily integrated into everyday life, and parents valued the sense of reassurance offered by the system. Parents’ ongoing struggle to balance control of their children with allowing independence was evident. For children who measured regularly, use appeared to reduce parental intrusions. For those who measured irregularly, however, parental reminders (eg, “nagging”) appeared to increase. Although increased reminders could be considered a positive outcome, they can potentially increase parent-child conflict and thus also undermine proper metabolic control. Parents felt that system appropriateness tapered off with the onset of adolescence, partly due to a potential sense of surveillance from the child’s perspective that could fuel oppositional behavior. Parental suggestions for further developments included similar alerts of irregular insulin dosages and automatically generated dietary and insulin dosage advice.

**Conclusions:**

User enthusiasm suggests that such systems might find a consumer market regardless of whether or not they ultimately improve health outcomes. Thus, more rigorous studies are warranted to inform guidelines for appropriate use. Potentially fruitful approaches include integrating such systems with theory-based parenting interventions and approaches that can aid in interpreting and responding to experiences of surveillance, virtual presence, and balances of power in e-mediated relationships.

## Introduction

Parents of children with type 1 diabetes are often involved in aspects of their children’s lives that they would ordinarily ignore [1]. Increased blood glucose monitoring is associated with improved glycemic control [2], which is essential in the prevention of serious, even life-threatening, complications of the disease. “Constant vigilance” is found to be a primary behavior strategy for parents coping with the responsibility of managing their child’s disease [3]. They are faced with the difficult balancing act of helping their child learn and conform to the rigorous blood glucose monitoring (BGM) and regulatory regimens, while at the same time allowing their child the trust and independence necessary for developing their own sense of autonomy and coping skills [4].

This challenge changes in character as children grow into adolescence, and adolescents report parental worry and intrusive behavior as a major source of conflict [5]. Heightened levels of conflict and low levels of family cohesion and support are associated with poorer metabolic control [1,6], poorer quality of life [7], and poorer adherence among adolescents [3,8]. Thus, conflict reduction and management is a major concern in diabetes care. Facilitation of appropriate parent involvement is also crucial since less parental involvement in diabetes care has been associated with poorer diabetes outcomes [4]. Interventions designed to enhance BGM but that inadvertently exacerbate parent-child conflict or undermine parent involvement may thus do more long-term harm than good.

Information and communication technologies (ICT) show promise as a platform for facilitating evidence-based disease prevention and self-management interventions [9]. Much of this work has centered on Internet applications that facilitate information, decision support, and social support [10-12]. These types of applications may be enhanced by adaptation to mobile platforms. As cell phones increasingly approximate personal computer (PC) functionality with Internet access, they become a highly accessible platform for facilitating a wide range of health interventions, some of which appear particularly promising [13,14]. Cell phone–based text messaging has become a socially popular form of communication, particularly among adolescents [15]. Cell phone usage is comparatively higher in Norway (site for study) than other Western countries (eg, 96% of Norwegians vs 60% of US population), with Norwegians sending an average of 68 text messages per month [16]. However, since the dispersion of cell phones is expected to be considerably higher than PCs, both in Western and developing countries [17], the potentials for dispersion of disease prevention and management support through mobile devices are considerable. Still, mobile technologies coupled with specific behavioral health strategies have yet to be utilized effectively [14].

The working hypothesis that guided design of the prototype and concept tested in this study was as follows: automatic transfer of measures from the child’s blood glucose meter to the parent’s mobile phone could ease parental worries and tendencies to intrude in their children’s lives by unnecessary reminders and/or questions. Potentially, this could, in turn, aid in decreasing levels of conflict in parent-child interaction and thus also increase adherence. While research into the psychosocial dynamics of mobile communications among adolescent peers is increasing [15], we are unaware of any studies that address these issues in the context of family disease management. In light of the critical role parent-child interactions play in the monitoring and regulation of blood glucose, this issue warranted particular attention in the formative stages of these applications. As argued by others [18], a qualitative approach was considered most appropriate in this phase. Also, since these types of self-management technologies may well be used by consumers without the supervision of health care professionals, it is crucial to tap into the perspectives of potential users as early as possible.

The aim of this exploratory pilot study was twofold. First, we sought initial user insights into the appropriateness of the concept, feasibility of use in daily life, and desired system functionality. Second, we sought indications of relevant approaches for future developments of monitoring and messaging systems in disease self-management. In this formative stage of development, our concern was to better understand the psychosocial issues potentially involved in the use of this type of technology in families. This is useful as a basis for designing systems, as well as the process and outcome studies that are ultimately needed for determining appropriate roles for mobile ICT in family health management.

## Methods

**Figure 1:**
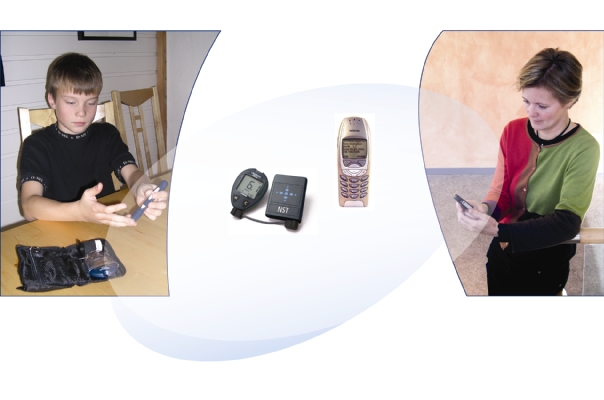
Blood glucose readings are sent to parent’s mobile phone

The prototype was developed at the Norwegian Centre for Telemedicine, a publicly owned non-profit national competence center. Using a Bluetooth connection, the prototype automatically transfers blood glucose readings from a blood glucose monitor (OneTouch Ultra from Lifescan) to a mobile phone after measurements are taken. The mobile phones (Nokia 7650) were programmed to automatically send the measurement results by means of the text messaging Short Message Service (SMS) to the parents’ mobile phone. The mobile phone sends the SMS without intervention from the user (in this case, the child) as long as the phone is within Bluetooth range (10 m) of the blood glucose monitor at the time of the blood glucose reading (Figure 1). When this range is exceeded, the blood glucose readings are sent in batches of five the next time the units are within range of each other.

Prior to the project, three pilot users tested the prototype for a period of 3 months, during which improvements were made. Based on this, 17 prototypes were developed, 15 of which were provided to the participants in the project and 2 of which were kept as backup and reference.

### Participants

Invitations to participate in the pilot study were sent to all 55 families of children with type 1 diabetes who, at the time, were being followed by the University Hospital of North Norway. The first 15 parent-child dyads (N = 30) who responded positively were accepted as participants (thus, we do not know how many nonresponders may have refused, had they been pursued). The group of children consisted of 11 boys and 4 girls, aged 9 to 15 years. The disproportionate number of boys may reflect a greater interest for technical trials among boys since gender distribution among the 55 approached parent-child dyads was fairly even. All of the children had received initial training courses for management of their diabetes, but they differed greatly in experience; duration of the disease ranged from 8 months to 6 years. For insulin injections, 13 reported using an insulin pen while 2 used an insulin pump. Before the trial, all of the parents and 11 of the children were frequent users of mobile phones, 3 of the children were nonfrequent users, and 1 child had not previously used a mobile phone.

### Procedure and Instruments

All parents signed an informed consent form on behalf of themselves and their children, and the intervention and methods were approved by the Regional Ethics Committee. The children were provided with the prototype-enhanced mobile phones, while all parents used their existing mobile phones to receive the SMS. They were trained in use of the system during a routine hospital visit and used it for approximately four months between October 2003 and February 2004. The participants were requested to use the system a minimum of three times a day, but they were reimbursed for the equivalent of 10 messages per day (approximately US$1.40) regardless of use. At their own cost, they were free to use the phone for private purposes during the trial. A diabetic nurse at the hospital handled any questions from users, channelling technical problems to the project manager.

At the completion of the trial, all 15 children and their parents completed separate questionnaires about use and satisfaction. A semi-structured guide for parent interviews was designed to elicit experiences and ideas regarding the potential benefits and pitfalls and further system developments. The interview posed open-ended questions addressing three overriding issues: stress and coping, the parent/child relationship, and system functionality. Parents were encouraged to freely share experiences and thoughts. All but one of the interviews were conducted over the phone. The interviewer, who was unacquainted with the parents, interviewed those parents that were available during a limited period, that is, 9 mothers and 1 father of children ranging in age from 9-14 years. After interviewing 10 parents, no new information emerged (“data saturation” [19]), and further interviews were deemed unnecessary for our preliminary purposes. Of the 10 interviews, 1 was lost due to a faulty audio recorder.

### Analysis and Presentation

The questionnaires were analyzed and reported as straightforward frequencies. For interviews, rigorous adherence to qualitative analysis procedures was deemed premature for our preliminary purposes, although efforts were made to comply with the basic principles of qualitative research [20]. Two co-authors (psychologists) independently read the transcripts, noting emerging themes and corresponding quotes that reoccurred across interviews thus allowing a broad range of possible interpretations and/or misunderstandings. These were then condensed to the nine themes presented as interview results. Presentations rely heavily on quotes from parents. These are edited to faithfully reflect what was said and meant, while at the same time ensuring readability [21].

## Results

The families used the system as requested, on average 3 to 4 times daily, mostly when the children were at school or away from home. As indicated by questionnaire responses in Table 1, both children and parents reported that automatic transfer of blood glucose measures was definitely a good thing (80% and 93%, respectively), that living with diabetes was at least to some extent easier (73% and 80%, respectively), and that the system helped parents feel reassured (100%). While children were split as to whether they wanted to decide themselves about sending glucose measures rather than having them sent automatically (40% yes and 53% no), most parents (93%) did not think children should make the decision to send measures.

**Table 1:** Questionnaire responses regarding child and parent satisfaction

**Questions to children (n = 15)**	**Yes, definitely (%)**	To some extent* (%)	**No, not at all (%)**
Was it positive that your parents received your blood glucose measures?	12 (80)	3 (20)	0
Did living with diabetes become easier with the system?	2 (13)	9 (60)	4 (27)
Would you like to decide yourself whether the blood glucose measure should be sent or not?	6 (40)	Undecided 1 (7)	8 (53)
**Questions to parents (n = 15)**			
Was it positive to receive the blood glucose measures from your child?	14 (93)	1 (7)	
Did it become easier to manage your child’s diabetes with the system?	6 (40)	6 (40)	3 (20)
Did receiving the blood glucose measure help you feel reassured?	14 (93)	1 (7)	0
Did receiving the blood glucose measures add to your worry?	1 (7)	1 (7)	13 (86)
Would you like for your child to decide whether the blood glucose measure should be sent or not?	1 (7)	Undecided 0	14 (93)

^*^ For two questions, the middle category was formulated as “undecided.”

The qualitative interviews among parents provided additional information and shed light on questionnaire responses. The nine interviews resulted in the following themes that reoccurred across interviews: (1) sense of security and reassurance, (2) nagging and scolding, (3) control, responsibility, and independence, (4) surveillance and opposition, (5) learning and age-phased appropriateness, (6) focus upon illness, (7) if it’s not automatic, forget it, (8) system type and functionality, and (9) it depends on how you use it.

### Sense of Security and Reassurance

The parents almost unanimously expressed appreciation for the security of knowing whether or not their children had measured their blood glucose and that they could intervene immediately if the values were alarming. Several expressed a wish to continue use. This was particularly apparent for newly diagnosed and younger children and when children were away from home or when the parent was traveling.

Both she and we slept better…. Without the system you can go around for hours saying to yourself, “What’s happened; What hasn’t happened; Should we call? No, maybe she’s measured.” It’s stupid to call and hassle her, you know. And then she’ll come home and hasn’t measured...while [during the trial] if we didn’t get an SMS we could just call right away.

When parents did not receive an SMS measure as expected, various interpretations were triggered. These could range from technical failure, to their child’s forgetfulness, to passiveness bordering on conscious sabotage on the part of their child. Parents describe this as a stress factor that had always been there on one level but was now dispersed throughout the day, as illustrated by two parents:

You didn’t [before the system] get stressed when you didn’t know she hadn’t measured. Now, the minute you expect it to come and it doesn’t, you start worrying, which maybe is negative, but not worse than you can live with.

I went around checking my phone all the time. I didn’t go with it in my pocket, but I checked my purse pretty often. That was pretty…what shall I say…but I guess it was tolerable.

This increased vigilance may help explain the feeling of one parent who responded in the questionnaire that the system added to worry. In general, both questionnaire and interview responses suggested that parents were more inclined to view the SMS message as reassuring.

### Nagging and Scolding

Some parents reported that their nagging increased, while others reported a decrease. This appeared to depend upon whether children monitored their blood glucose regularly. For example, one parent who received the updates on her child’s blood sugar regularly reported that

I feel safer, so I pester him less.

In contrast, another parent whose child did not monitor regularly reported that

Maybe I nag and scold more after seeing how negligent he can be…. I call him at school and say, “Why haven’t you measured as we agreed?” which I’m sure annoys him…. Yes, I’m worse after we started with this system…. But, as I tell him, it’s for his own good…. He can avoid calls from me by remembering to measure.

Several parents described making conscientious efforts to remind their children in ways that would not be perceived as nagging, for example, by finding other excuses to call and thus triggering measures indirectly.

### Control, Responsibility, and Independence

Regardless of system use, parents described their struggle to find a balance between the control they felt necessary to ensure the health of their child, while at the same time allowing for the child to develop their own sense of responsibility and independence. System use appeared to tip the balance in both directions. While one informant thought “…it clearly placed more responsibility with the child,” another said, “It’s obvious that we’re the ones that have increased our responsibility.”

Those who felt that their children’s responsibility was enhanced through use argued that they were provided with a safe framework within which the child could learn to make their own decisions with parental guidance.

He can feel secure knowing that others are part of this and see, but that he can figure things out and do his own thing anyway.

Another noted

There were a couple of times I forgot my phone, so he called me wondering whether or not I’d seen his measures, and if he should take something. So I think it worked well.

Those who felt that the parents’ responsibility was increased sensed that their child could be pacified. One parent described her child’s likely thought process:

I don’t need to bother [measuring], since Mom will start calling to hassle me pretty soon, and then I can do it.

This parent was nevertheless uncertain about the degree to which the system was to blame, since her child was “sick and tired” of her illness long before the system was introduced. As she put it,

It could well be that the system undermines children’s responsibility for their illness, but during the period we used it…in the situation she was in…it was a help for us.

One parent alluded to a distant form of presence.

She always takes more responsibility when she’s alone, than when she’s with us.

The meaning the system had for this child in this respect was, however, unclear for the parent.

She knew it happened [that parents saw her measures], but it happened so automatically, I don’t think she thought about it.... At least it didn’t pacify her.

### Surveillance and Opposition

One concern is that the system may create a negative sense of surveillance and thus fuel oppositional behavior. For older children, and those with preexisting levels of conflict, the system appeared to represent an additional source of tension.

This parent related episodes before the trial where their boy had consciously deceived them:

Of course we’ve wondered if he’d measure a buddy rather than get hassled by us. So who knows? But I don’t think he’s done it [sent a buddy’s blood glucose reading].

Another parent alluded to the possibility of her child consciously refraining from measuring when she knew her measures would trigger a reaction from the parent.

If she doesn’t want me to know she’s high or low, she’ll refrain from measuring. She’d know she’d get a message or phone from me, but she could elude me anyway by not taking the phone or just turning it off. So she escapes me regardless.

Parents differed in the degree to which they reported discussing this issue with their child.

### Learning and Age-Phased Appropriateness

Parents indicated that the potential of the system to facilitate knowledge and skills about BGM and regulation was greatest at the onset of disease. Parents were also fairly consistent in their view that the appropriateness of the system tapered off with the onset of adolescence. One indicated that the system would have been particularly useful during the period when the child was newly diagnosed and in preschool.

However, parents differed in their perceptions of when the child became too old for the system. A parent with a 14-year-old stated

He hasn’t really taken much responsibility himself…. I feel he’s a little too young.... He’s quite good at following up, but I still prefer to be in control…so this [system] has been positive.

Another parent with a younger child said

The messages are positive for us [parents]…for our reassurance. But I don’t feel it’s right for him who’s 11 years old and should manage himself. It gets too controlling, I think. But he’ll have to speak for himself.

Several parents appeared uncertain about age-appropriate expectations of their children, suggesting that it may be helpful to assist families in sharing experiences about how other parents deal with this issue.

### Focus on Illness

Can system use exacerbate or reduce the dominance of illness as the focus of parent-child interaction? The parents who explicitly commented on this tended to think that their focus on illness was the same or less but that this depended on the way it was used.

One parent didn’t think it made any difference since they had no choice but to constantly focus on the illness:

We have to hang over her all the time anyway or else things would fall apart. The system just helped us to follow up, it didn’t make us more focused [on illness] than we were already.

Another appreciated the system because it allowed focusing on other issues in their relationship:

It was wonderful! We started talking about other things—you know, mother-child dialogue—not blood sugar and setting insulin that had been the main content of our communication for a period, which was bad. It was absolutely fantastic, especially in the beginning [of the trial] when we noticed it so clearly. And I think he experienced it too.

This parent underlined the importance of not responding to every SMS, which could trigger an unnecessary focus on disease.

### If It’s Not Automatic, Forget It

Parents were adamant about the measures needing to be transferred automatically, and they used expressions like “Alpha Omega,” “extremely important,” and “very thankful for it” to underline their view. They had no faith in a concept requiring a conscious effort on the part of their kids to trigger the transfer of measures to parents, arguing that they had enough to remember as it was. Also, although schools made exceptions to the mobile phone ban in class for these children, it was appreciated that the phones could remain unhandled and hidden in their bags.

### Suggested Functionality

Suggestions for improving functionality included automatically generated dietary and insulin dosage advice. One parent argued for developing the same concept for the insulin unit, but only for transmitting irregular doses. One time his child had taken 20 insulin units in response to high blood sugar, but a few minutes later, he forgot he had done it and set 20 more, sending him into a coma.

If we’d known, we could have prevented him getting so traumatized…. These cases are about life and death.

None of the parents thought the system would be useful in interaction with their health care provider, except if automatically generated historical graphics could be transferred to their provider in preparation for their ordinary quarterly checkups.

### Depends on How You Use it

In varying ways, parents indicated that it was more the way they used the system than the system itself that was important. For example, one parent underlined how routines for system use could limit the dominance of the system in interaction with her child.

I think it’s very important that you have it on all the time, even though it isn’t economically smart. You get a message, and you just think “OK.” Of course, if one talks about every single message, or sends a response, that’s different. It [degree of interference by the system] has to do with how one uses the information and the system.

## Discussion

The ultimate objective of the system tested is to improve health and quality of life by supporting daily blood glucose monitoring and regulation processes in families. In this study, we sought an understanding of the tested system’s potential role, feasibility of use in daily life, desired functionality, and approaches that may be relevant for future developments and research.

### Potentials and Concerns

Knowledge of their children’s blood sugar status eased parental concerns. This appeared to lessen parental intrusions in those cases where children measured regularly. For those who measured irregularly, parental reminders (eg, “nagging”) appeared to increase. While an increase in parent reminders could be considered a positive outcome in light of studies underlining the importance of parent involvement in monitoring [2,4], it must be acknowledged that this can potentially increase parent-child conflict, particularly among adolescents and those with existing tensions. Discrepancies between parents and children as to whether or not the measures should be automatic underscore the need for more in-depth inclusion of children’s perspectives in future studies.

Some parental responses could be construed to suggest that receiving automatic measures throughout the day could be experienced as an invasion in their lives that they would rather be without, but that they could not or would not admit it (“*…*but I guess it’s tolerable,” “...but it’s not worse than you can live with”). Obviously, the disease itself is an unwanted “invasion” both in the lives of their children and themselves. Presumably, parents feel morally obligated to tolerate whatever is necessary to ensure the health of their child, thus denying themselves more “egoistical” reactions. If the virtual presence of children (through expectations of regular SMS messages demanding attention) exacerbates the existing burden of care among parents, it could inadvertently undermine their long-term involvement and/or fuel parent-child conflict. The concept of “constant vigilance” [3] associated with the burden of diabetes care may be useful in further exploring how system use may add to or relieve the burden of care. This should also include other caretakers’ (eg, family, pre-school and school staff) and how system use may influence their willingness to accept responsibility for care.

### Concept Suitability and System Functionality

Parents suggested that the system might be most beneficial for those who are younger and newly diagnosed. Our pilot users were aged 9 to 15, having lived with the diagnosis of diabetes from 8 months to 6 years, thus limiting our ability to shed light on the appropriateness for younger (eg, pre-school) children. One study found that mothers with pre-school children claim lack of confidence in other caregivers as a major reason for not having placed their children in day care [3]. The possibility that system use could support involvement of other caregivers in relieving the burden of care for parents of younger children may be worth pursuing.

Suggested functionality included a similar concept for alerting parents of irregular insulin dosages, as well as automatically generated dietary and insulin dosage advice. Combinations of information, decision support, and social support are core elements of existing Internet-based self-management applications [10,12]. Merging these types of applications with mobile monitoring and messaging functionality may enable relevant support whenever and wherever needed [14]. By building on daily life technologies (eg, blood glucose meters and mobile phones) the threshold for use should be relatively low, as our study suggests.

Historical graphs of measures were also suggested. We are currently working to enable such graphs, which may be used in conjunction with follow-up consultations. This can enhance both system evaluation and give health care providers an opportunity to supervise system use.

### Future Developments and Research

The enthusiasm expressed by interviewed parents is worth noting, despite the weaknesses of our sample (see below). It may very well be that such systems find a consumer market regardless of whether or not they ultimately succeed in improving health outcomes. Parents of children with chronic health problems can face “life and death” issues, as one parent put it. Some may find that relief from some of the worries associated with chronically ill children is sufficient motivation for system use, even if the way they use it inadvertently exacerbates family tensions, and possibly also blood glucose control. This provides all the more reason to take the technology seriously and pursue more rigorous approaches to development and evaluation. As is typical of eHealth innovations [9], we are nowhere near reliable answers to very basic questions such as for whom, under what conditions, and how monitoring and messaging systems may relate to health outcomes.

Broader perspectives, two of which are suggested here, may be helpful in guiding future developments and evaluations. First, it may be useful to view these systems as an element of parenting, rather than a simple monitoring and messaging device. As illustrated by the parents in our study and others [1,4,8], the ordinary challenges of parenting are compounded when children have a chronic disease. Our findings suggest that system use may merely intensify ongoing parent-child interaction patterns—for better or worse—unless incorporated into conscientious efforts to improve such patterns. There is a growing body of evidence that behavioral family intervention approaches are effective at reducing parent-child conflict, increasing parents’ competence and monitoring skills, and increasing child compliance, including families whose children have chronic health problems[22,23]. As such, evidence-based approaches to parenting practices may be useful in informing both system design choices and guidelines for use. These approaches incorporate various types of counseling and social support, which we believe our pilot users could have benefited from in conjunction with system use. As technologies such as those described in this paper become more available, it would seem important for health care providers to introduce them as an element in more holistic, empirically supported approaches to family interaction, coping skills, and child-rearing practices in disease self-management.

Another perspective suggested for guiding further developments relates to the dilemmas faced when introducing technology into interpersonal (eg, child-parent, doctor-patient) relationships. The parents in our study voiced some of these dilemmas. On the one hand, they valued the security of knowing that they could intervene if their child’s blood glucose values were alarming. At the same time, they expressed concerns that the system could inadvertently undermine the child’s independence and confidence in their own coping skills and that a sense of surveillance might fuel oppositional behavior, particularly among adolescents. One potentially fruitful approach to such dilemmas is outlined in Spears and Lea’s SIDE (Social Identity and Deindividuation) model [24]. This model of computer-mediated communication suggests how we might anticipate and interpret experiences of surveillance, virtual presence, and balances of power in e-mediated interpersonal relationships. They remind us of the need to critically examine assumptions about the role of technology and the implications of use.

### Study Limitations

The limitations of this study (beyond our exploratory and formative purposes) are obvious. Our sample is quite possibly biased since it is not random. Those who responded first to our invitation may, for example, be particularly technology savvy. The experiences of the five parents (one-third of pilot users) who were not interviewed may have deviated consistently from our respondents, although we have no reason to believe this was the case. However, even if the last five were all negative or all positive, our conclusions would remain the same.

### Conclusion

The mobile monitoring and messaging system tested is feasible to use in daily life. While system use eases parental worries, and as such shows potential as an aid in disease self-management, it is unclear if or how this will improve child-parent interactions and/or health outcomes. User enthusiasm suggests that such systems might find a consumer market regardless of whether or not they ultimately improve health outcomes. Thus, more rigorous studies are warranted to clarify these issues and inform appropriate use. Potentially fruitful approaches include integrating such systems with theory-based parenting interventions and approaches that can aid in interpreting and responding to experiences of surveillance, virtual presence, and balances of power in e-mediated relationships.
